# Differential cortical responses of robot-assisted active and mirror therapy task conditions in stroke patients and healthy controls: a comparative fNIRS study

**DOI:** 10.3389/fneur.2026.1766545

**Published:** 2026-06-18

**Authors:** Changhao Le, Huamin Li, Jun Zhou, Jia Fu, Xing Wen, Xiarong Huang, Huali Tang, Mengjian Qu

**Affiliations:** 1 Department of Rehabilitation, Hengyang Medical School, the First Affiliated Hospital of University of South China, Hengyang, Hunan, China; 2 Rehabilitation Laboratory, Hengyang Medical School, the First Affiliated Hospital of University of South China, Hengyang, Hunan, China; 3 Department of Rehabilitation Sciences, The Hong Kong Polytechnic University, Kowloon, Hong Kong SAR, China

**Keywords:** active movement mode, cortical response, functional near-infrared spectroscopy, mirror therapy mode, robot-assisted task, stroke rehabilitation

## Abstract

**Background:**

Robot-assisted task conditions incorporating Active Movement Mode (AMM) and Mirror Therapy Mode (MTM) is widely used for upper-limb stroke rehabilitation. However, their differential task-related cortical responses remain insufficiently characterized.

**Objective:**

This study aimed to use functional near-infrared spectroscopy (fNIRS) to characterize and compare the task-dependent cortical activation and rest state functional connectivity profiles elicited by AMM and MTM in stroke patients and healthy controls.

**Methods:**

Twenty-three chronic stroke patients and twenty-three matched healthy controls performed robot-assisted upper limb tasks in both AMM and MTM. Cortical activation (Beta value) in the prefrontal (PFC), primary motor (M1), supplementary motor (SMA), premotor (PMC), and primary somatosensory (PSC) cortices was measured using a 48-channel fNIRS system. A Two-way repeated measures ANOVA to compare cortical activation; Hemispheric activation distribution was assessed by calculating the lateralization index (LI). To evaluate brain-behavior relationships, we used Pearson or Spearman correlation analyses to examine the association between ROI activation levels and FMA-UE scores. Furthermore, resting-state functional connectivity (RSFC) was compared between eyes-open and eyes-closed conditions. All multiple comparisons were corrected using the Benjamini-Hochberg FDR method.

**Results:**

Both groups showed extensive cortical activation across both modes ( p<0.01 ). Analysis of variance indicated significant main effects of task in the ipsilesional PFC and PSC, along with the contralesional PFC and PMC ( p<0.05 ), where activation in the AMM mode was consistently higher than in the MTM mode. Resting-state analysis revealed that stroke patients had significantly weaker inter-hemispheric and intra-hemispheric RSFC in the eye-open (EO) condition than healthy controls ( p<0.05 ). No significant correlations between brain activity and behavior or lateralization effects were found.

**Conclusion:**

Active Movement Mode and Mirror Therapy Mode exhibited distinct cortical activation and functional connectivity profiles during robot-assisted upper-limb tasks. Compared with MTM, AMM was associated with stronger recruitment of several motor-related regions, while differences in connectivity further supported distinct modes of neural engagement.

**Clinical trial registration:**

Unique identifier: ChiCTR2500096992, URL: https://www.chictr.org.cn.

## 1 Introduction

Stroke remains a leading cause of long-term disability worldwide (1), with upper limb motor impairment affecting >50% of survivors and severely compromising activities of daily living (ADL) such as feeding, dressing, and grooming (2). Exercise therapy is frequently employed as a crucial tool to assist patients in regaining physical function, which is a fundamental ingredient of neural rehabilitation (3).

Based on the mode of execution, motor therapy can be categorized into several types, including active, active-assisted, passive, and resistive modes (4). Physical therapists typically match the appropriate training method to a patient’s residual function and needs. Among these, active movement requires patients to execute movements voluntarily, primarily targeting muscle strength, endurance, and motor coordination, making it a cornerstone of clinical practice (5). In addition to conventional motor therapies, mirror therapy is an evidence-based modality widely used to improve upper limb function. During mirror therapy, participants are instructed to perform task-oriented movement with one hand (typically the unaffected hand) while observing the reflection of this moving hand in a mirror placed on their midsagittal plane. This approach has been proven effective in promoting the motor recovery of the paretic upper limb in stroke patients (6, 7). Technological advancements have transformed the delivery of clinical practice. Robot-assisted task (RAT) has now integrated both of the aforementioned therapeutic approaches and is frequently applied in clinical settings (8). Active RAT requires patients to exert volitional effort to initiate and execute movements, while the robotic device provides adaptive support proportional to their residual motor abilities (9). Qiao et al. combined a robotic glove with mirror therapy to improve upper limb motor function in stroke patients, finding the approach effective in enhancing paretic limb motor function and ADL for patients in the subacute stage (10). Importantly, AMM and MTM should not be regarded as equivalent task conditions. AMM involves more direct voluntary effort and self-generated movement of the paretic limb, whereas MTM depends more heavily on mirrored visual feedback and movement of the non-paretic side. In clinical practice, both training modes are widely employed. However, their selection by therapists is often empirical, lacking objective criteria based on underlying neural mechanisms. The Active Movement Mode (AMM) demands volitional effort from the patient and may directly engage the damaged corticospinal tract. In contrast, the Mirror Therapy Mode (MTM) utilizes visual feedback to bypass the lesioned hemisphere and activate the mirror neuron system.

Previous neurophysiological studies suggest that upper-limb active movement is commonly associated with activation in distributed motor-related cortical regions, including the primary motor cortex (M1), supplementary motor area (SMA), premotor cortex (PMC), and primary somatosensory cortex (PSC). In a stroke-related fNIRS study, Xia et al. reported that active upper-limb movement elicited stronger cortical activation than passive movement, and that additional visual performance feedback was associated with greater activation in motor-related regions, particularly in the hemisphere contralateral to the moving limb, including the PMC and SMA (11). In healthy adults, Yang et al. similarly observed generalized bilateral activation during upper-limb movement, with greater activation in the primary motor cortex contralateral to the moving limb (12). By contrast, studies of mirror visual feedback and mirror therapy have implicated cortical networks involved in action observation, sensorimotor integration, and visuospatial attention. Using fNIRS in healthy participants, Bai et al. found that mirror visual feedback was associated with higher activation in the ipsilateral SMA and superior parietal lobule, whereas effects in the ipsilateral sensorimotor cortex were comparatively modest (7). In stroke patients, Michielsen et al. reported with fMRI that mirror-induced visual illusion during bimanual movement increased activity in the precuneus and posterior cingulate cortex, while mirror-related activity in motor or mirror-neuron-system regions was not observed (13). Taken together, these findings suggest that active and mirror-based paradigms may engage overlapping but non-identical cortical networks. However, reported activation patterns vary across studies, likely because of differences in participant characteristics, task structure, movement generation, and feedback modality.

At the same time, it is important to recognize that AMM and MTM differ along several task dimensions beyond the mode label alone. Compared with MTM, AMM typically involves greater voluntary effort by the paretic limb, stronger self-generated motor control, higher action agency, and, in some implementations, additional resistance. In contrast, MTM places greater emphasis on visual feedback and mirrored movement driven by the non-paretic side. Accordingly, differences in cortical activation between these paradigms cannot be attributed to a single isolated mechanism. Rather, they are more appropriately understood as paradigm-related neurophysiological differences arising from the combined influence of effort, resistance, movement generation, feedback modality, and action agency. Moreover, these paradigms have often been studied separately rather than compared directly within a unified robot-assisted framework, especially in studies including both stroke patients and healthy controls.

Functional near-infrared spectroscopy (fNIRS) provides a practical and noninvasive method for examining cortical hemodynamic responses during movement tasks. Because it is relatively tolerant of motion and suitable for task-based assessment in rehabilitation settings, fNIRS is well suited for comparing cortical activation patterns elicited by different robot-assisted task paradigms. In addition, including healthy controls alongside stroke patients may help distinguish stroke-related alterations in activation and connectivity from patterns observed in the intact motor system.

Therefore, the primary aim of this study was to characterize and compare task-dependent cortical activation patterns elicited by robot-assisted AMM and MTM in stroke patients and age-matched healthy controls using fNIRS. Specifically, we examined regional cortical activation across predefined motor-related regions of interest, together with hemispheric lateralization and resting-state functional connectivity. We hypothesized that: (1) AMM and MTM would elicit different cortical activation profiles; (2) AMM would be associated with stronger activation in regions related to voluntary motor control and attentional demand, particularly the PFC and sensorimotor regions; and (3) compared with healthy controls, stroke patients would show altered task-related activation patterns and weaker resting-state functional connectivity.

## 2 Materials and methods

### 2.1 Participants

This study included 46 participants (32 males and 14 females). Participants were included if they presented with their first unilateral stroke (confirmed by CT/MRI), exhibited moderate upper limb impairment (Functional Test for the Hemiplegic Upper Extremity, Hong Kong version [FTHUE-HK] score: 3–6), and possessed adequate cognitive capacity to follow instructions (Mini-Mental State Examination [MMSE] score ≥20). All participants were right-handed prior to the stroke. Exclusion criteria were any history of other neurological or psychiatric conditions, severe cognitive or aphasic deficits, or contraindications to fNIRS measurements. Healthy controls were individually matched with patients based on age and sex, and were free from other severe neurological, somatic, or psychiatric disorders. They refrained from taking any medications known to affect cortical excitability prior to testing and avoided consuming coffee, tea, or other substances that might lower the cortical excitation threshold. This study is a single-center, observational, cross-sectional controlled study. Stroke participants will be recruited from inpatients or outpatients at the Rehabilitation Medicine Center of the First Affiliated Hospital of the University of South China, while healthy controls will be recruited from relevant hospital staff and community volunteers. All participants will attend only one laboratory visit and undergo fNIRS assessment under standardized robot-assisted upper-limb task conditions. The study does not involve treatment allocation, treatment-course design, or follow-up of therapeutic outcomes. Patients performed the two upper limb robot task conditions using their hemiplegic limb in both AMM and MTM. Healthy controls will be recruited from relevant hospital staff and community volunteers to establish a candidate pool of healthy controls who meet the inclusion and exclusion criteria. After enrollment of the patient group is completed, healthy controls matched to the patients on variables such as age, sex, and handedness will be selected from the candidate pool according to prespecified matching principles for inclusion in the final analysis. In this study, healthy controls serve as a normative reference baseline to identify post-stroke cortical activation patterns that deviate from healthy cortical dynamics, including aberrant lateralization and hypoconnectivity. Participants provided written informed consent after being fully informed about the study procedures. The study protocol was approved by the Ethics Committee of the First Affiliated Hospital of University of South China (2024KS-KF-28-02) and registered with the Chinese Clinical Trial Registry (ChiCTR2500096992).

### 2.2 Upper limb rehabilitation robot

**Figure 1:**
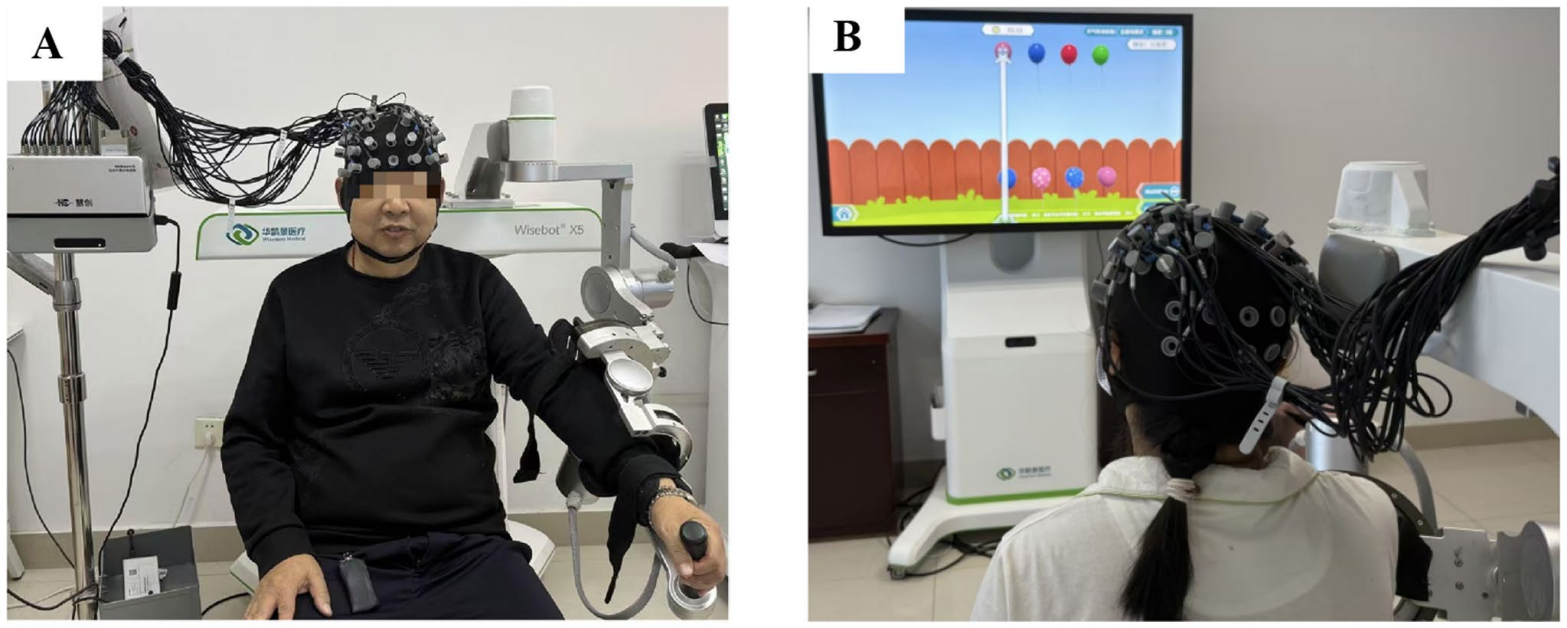
**(A)** Rehabilitation robot and fNIRS equipment. **(B)** experimental scene of subjects.

This study utilized the Wisebot X5, a three-dimensional (3D) upper limb exoskeleton rehabilitation robot developed by Shenzhen Wisemen Medical Technologies Co., Ltd. (Shenzhen, China), to implement passive, active, and mirror therapy task conditions. As an exoskeleton-type device, the robot’s structure is designed to align with the user’s arm, enabling multi-joint movement within a 3D workspace that simulates activities of daily living. The system is equipped with precise force sensors and a depth-sensing camera and delivers real-time visual and auditory feedback through immersive, gamified 3D scenarios (Figure 1A).

During the sessions, subjects were instructed to perform functional, task-oriented exercises displayed on the screen (Figure 1B). The visual prompts, intended trajectory, and difficulty level of these tasks were standardized for all participants. In the passive mode, the robot guided the subject’s impaired limb through the entire range of motion at a constant, pre-defined speed. In the active mode, subjects independently initiated and completed the movements to achieve the task goals. For the mirror therapy mode, the depth-sensing camera captured the real-time motion of the subject’s unimpaired arm, which the robotic exoskeleton then simultaneously mirrored to guide the impaired arm through the same movements. While these movement patterns and visual instructions were standardized across modalities, it should be noted that the actual task demands—including physiological resistance and volitional drive—varied according to the specific exercise mode and individual patient performance. This approach ensured procedural consistency while allowing for the inherent mechanical and neural differences between active and mirror-assisted task conditions.

### 2.3 fNIRS data acquisition

**Figure 2:**
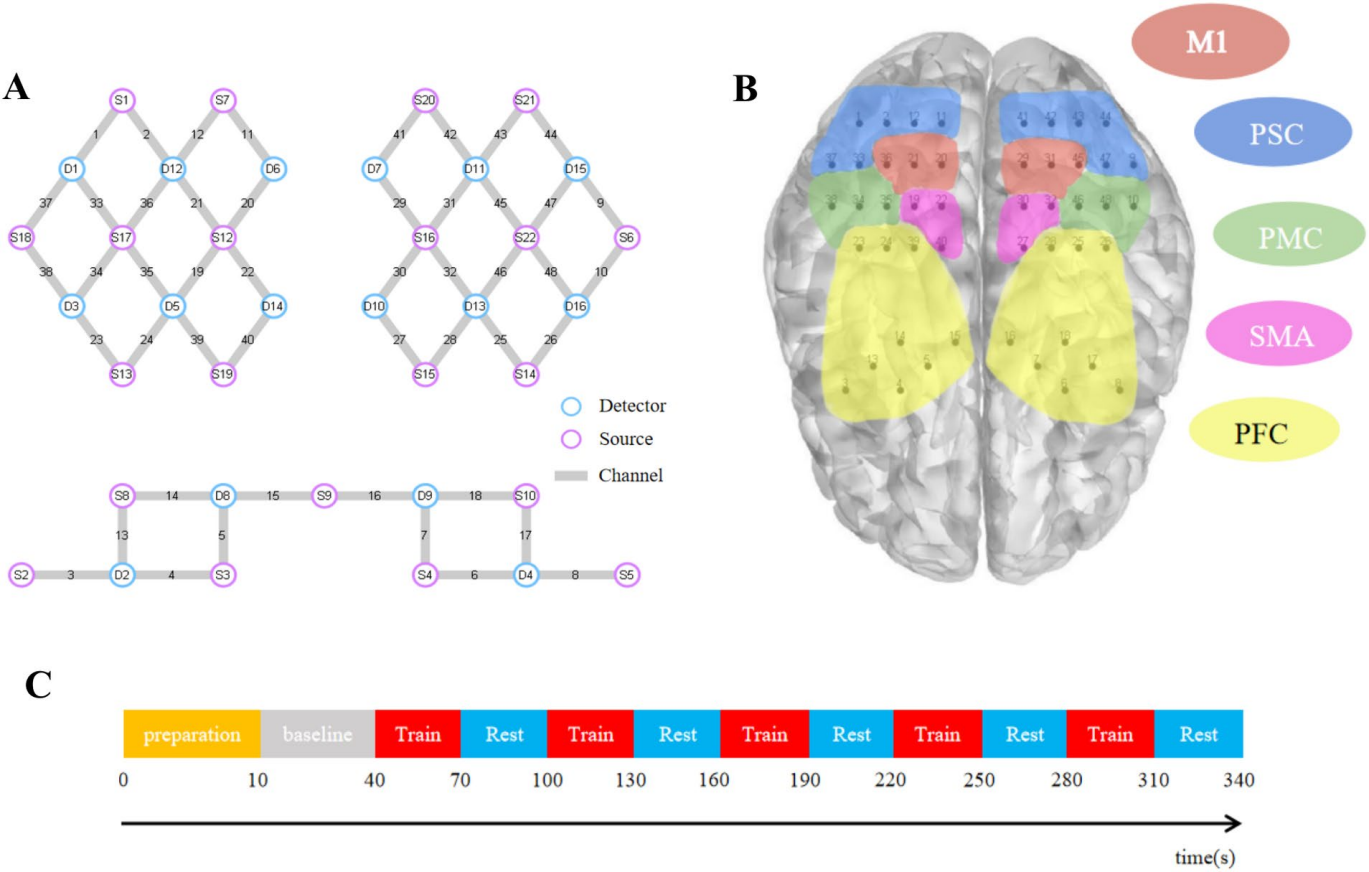
**(A)** Arrangement of fNIRS channels; **(B)** Elaboration of regions of interests (ROI) based on Brodmann area. M1: primary motor cortex; PSC: primary sensorimotor cortex; PMC: pre-motor cortex; SMA: supplementary motor area; PFC: prefrontal cortex; **(C)** fNIRS block design.

Cortical activity was continuously monitored using a fNIRS system (NirSmart-3000A, Danyang Huichuang Medical Equipment Co., Ltd., China). This system employs continuous-wave light-emitting diodes (LEDs) with wavelengths of 730 nm and 850 nm, operating at a sampling rate of 11 Hz. A 48-channel system was constructed by configuring 37 NIRS optodes (21 sources and 16 detectors) in a rectangular grid pattern (Figure 2A). These optodes were securely mounted within a specialized cap. To ensure precise positioning and consistency across measurements, specific anatomical landmarks on the scalp surface were carefully measured after cap placement, along lines extending from the nasion to the inion and connecting the left and right preauricular points. This ensured alignment of the cap’s Cz electrode position with the corresponding anatomical location (14). Before starting the task, the assessor positioned the optode cap on the participant’s head, aligning its central point with the intersection of the interauricular line and the nasion-inion line (Cz point). Contact pressure for each optode was adjusted to ensure good fNIRS signal quality across all channels. Pre-acquisition was performed before formal data collection to observe channel data quality and make adjustments, followed by software-based automatic gain adjustment for each channel. The experiment commenced once stable fNIRS signals were obtained.

The PFC, M1, SMA, PMC, and PSC were selected as Regions of Interest (ROIs) in this study as they collectively constitute the core neural network for motor planning, execution, sensory feedback, and higher-order cognitive control (15, 16) (Figure 2B). Specifically, M1 serves as the final output pathway for voluntary movements and is essential for controlling the upper limbs; SMA and PMC, play pivotal roles in the planning, sequencing, and preparation of movement. The SMA is primarily associated with internally-driven, complex motor sequences, while the PMC is more involved in movements guided by external stimuli, such as visual cues. Furthermore, the PMC is a key node in the mirror neuron system. PSC processes proprioceptive and tactile feedback from the moving limb, this sensory input is crucial for real-time adjustment and optimization of motor control and also plays a role in motor learning and functional remodeling. Finally, the PFC is engaged in higher-level cognitive processes such as attentional allocation, goal setting, and motor inhibition. The correspondence between the ROIs and the 48 fNIRS channels is provided in the Table 1. The arrangement of optodes relative to these ROIs is depicted in Figure 2.

**Table 1:** Correspondence between the ROIs and the fNIRS channels.

ROI	Channel
Left PFC	16,18,6,7,17,8,28,25,26
Right PFC	3,4,5,13,14,15,23,24,39
Left M1	29,31,45
Right M1	20,21,36
Left SMA	30,32,27
Right SMA	19,22,40
Left PMC	10,48,46
Right PMC	38,34,35
Left PSC	9,47,44,43,42,21
Right PSC	37,33,1,2,12,11

ROI: region of interest; PFC: prefrontal cortex; M1: primary motor cortex; SMA: supplementary motor area; PMC: premotor cortex; PSC: primary somatosensory cortex.

### 2.4 Study protocol

An upper limb motor task, with a total duration of 330 s divided into 5 blocks, was performed. Each block consisted of alternating 30-s movement periods and 30-s rest periods. A 30-s baseline data acquisition period was included before the first block commenced, serving as a reference for subsequent brain activation levels during movement states. Prior to the blocks, there is a 10-s preparation period for subjects (Figure 2C).

Both the modes were centered on a “Sorting items” task where patients used their impaired limb to select the correct item from a shelf and deposit it into a bag following system-generated prompts. In AMM, patients were required to use their paretic upper limb to perform the task. Three levels of resistance were available (1 kg, 2 kg, and 3 kg), which were selected based on the patient’s ability to facilitate maximum engagement of their residual capabilities. While in MTM, patients had to control the unaffected hand via the robotic arm to sort items into bags, which was unresisted. The other robotic arm drove the movement of the impaired hand, which also moved without resistance. Across both conditions, the maximum workspace was capped at a range of approximately 90° horizontal abduction to 40° horizontal adduction, and 45° to 130° flexion at the shoulder. The effective ROM was tailored for each patient based on their pre-assessed passive ROM and their specific movement trajectories during the game. The maximum velocity of the arm was limited to 60°/s, with the actual speed being patient-determined. These discrepancies are an inherent consequence of the distinct theoretical foundations underpinning each modality; therefore, perfect equivalence between them could not be ensured. Prior to the experiment, the assessor instructed participants to familiarize themselves with the robot operation and experimental procedures. During the experiment, participants were instructed to remain as relaxed as possible, minimize facial and head movements, and move only the tested unilateral or bilateral arm. During rest periods, participants were prompted to remain still (head, trunk, limbs), avoid talking and making facial expressions, and maintain steady breathing. Accordingly, the comparison should be interpreted as a contrast between two clinically implemented paradigms rather than a single-factor mechanistic comparison. Observed differences may reflect the combined influence of movement generation, resistance, action agency, and feedback modality.

### 2.5 fNIRS data process

Collected fNIRS data underwent quality control procedures. Data quality assessment and analysis were performed using Nirspark software. First, channels with blank signals were identified and excluded. Subsequently, channels exhibiting clearly identifiable physiological waveforms (e.g., cardiac pulsation) in the frequency spectrum and devoid of uncorrectable artifacts were selected for further analysis. Raw optical data were converted into changes in oxygenated hemoglobin concentration (ΔHbO) based on the modified Beer–Lambert law (17). Data were then preprocessed, including motion correction using the channel and temporal derivative method (STD: 6, windows percent: 0.5) and band-pass filtering (0.01–0.2 Hz) (18). Preprocessed data were block-averaged, with epochs defined from 5 s before task onset to the end of each individual block. Block-averaged data were used to directly compute feature values such as mean amplitude, slope, and difference. Furthermore, these data were used for estimation within a General Linear Model (GLM) framework.

Preprocessing for GLM analysis included Gaussian smoothing with a 4-s full width at half maximum (FWHM) kernel to correct for short-term serial correlations, and wavelet-minimal description length (MDL) detrending with a 128-s period length to remove low-frequency drift (19). These parameters were incorporated into a GLM based on experimental conditions, with ΔHbO as the dependent variable. The GLM was estimated using a Statistical Parametric Mapping (SPM) algorithm (20).

RSFC analysis was conducted using the Network module within the NirSpark software (Danyang Huichuang Medical Equipment Co., Ltd., China). For each channel (CH) pair, Pearson correlation coefficients were calculated between oxygenated hemoglobin concentration time series and subsequently normalized into *z*-values via Fisher’s *r*-to-*z* transformation to ensure normality. Finally, the resulting 55 × 55 FC matrices were averaged to quantify functional connections.

### 2.6 Statistical analysis

All statistical procedures were performed using Python, with the significance threshold set at *α* = 0.05.

#### 2.6.1 Cortical activation analysis

To assess cortical activation patterns across groups and motor paradigms, the mean beta value within predefined ROIs served as the dependent variable. Data normality was verified using the Shapiro–Wilk test. A 2 × 2 two-way repeated-measures ANOVA was then conducted, with Group (Stroke Patients vs. Healthy Controls) as the between-subject factor and Task Mode (AMM vs. MTM) as the within-subject factor. This model evaluated both main effects and their interaction. Where significant effects were identified, post-hoc pairwise comparisons were performed.

#### 2.6.2 Brain-behavior correlation analysis

To examine the relationship between cortical activation and upper limb motor recovery, correlation analyses were conducted between ROI-specific activation values (beta value) in the patient group and their corresponding FMA-UE scores. Pearson correlation coefficients were calculated for normally distributed data, while Spearman rank correlation was used for non-normal distributions.

#### 2.6.3 Laterality analysis

The hemispheric distribution of brain activation was quantified using the lateralization index (LI), calculated from the mean activation values of each ROI.

$$\mathrm{LI}=\frac{\left(\text{ipsi}-\text{contra}\right)}{\left(|\text{ipsi}|+|\text{contra}|\right)}$$

To determine whether specific regions exhibited significant lateralization, LI values for both groups and modes were compared against zero using either one-sample *t*-tests or Wilcoxon signed-rank tests, depending on the data distribution.

#### 2.6.4 Resting-state functional connectivity analysis

FC matrices were constructed for both eyes-closed (EC) and eyes-open (EO) resting states. Pearson correlation coefficients were calculated between all channel pairs and normalized via Fisher’s *z*-transformation. Differences in connectivity strength between stroke patients and healthy controls were evaluated using independent samples *t*-tests or Mann–Whitney *U* tests as appropriate.

#### 2.6.5 Multiple comparison correction

To account for Type I error accumulation arising from multiple ROI and condition comparisons, all *p*-values were adjusted using the Benjamini-Hochberg False Discovery Rate (FDR) procedure. Results were considered statistically significant when the corrected *p* < 0.05.

## 3 Results

### 3.1 Demographic and clinical characteristics

This study enrolled 46 right-handed subjects, consisting of 32 men and 14 women with an average age of 52.5 years. The cohort was organized into 23 pairs, with each patient matched to a healthy control subject based on age and gender. There were no significant statistical differences between patients and controls in terms of the age or gender (Table 2). This table also provides detailed clinical characteristics of the patients, including duration of stroke, stroke type, lesion location, and scores on FMA-UE.

**Table 2:** Demographic characteristics.

Demographic data	Patients (*N* = 23)	Controls (*N* = 23)	*p* value
Gender (male/female)	16/7	16/7	1.00
Age (mean ± SD)	52.4 (12.7)	52.5 (11.3)	0.941
Duration (mean ± SE)	388.72 (193.47)	/	/
Type of stroke (1/2/3)	14/8/1	/	/
Lesion location (a/b/c)	0/22/1	/	/
FMA-UE (mean ± SD)	28.49 (13.82)	/	/

Type of stroke includes cerebral infarction (1), cerebral hemorrhage (2), Hemorrhagicinfarction (3); Lesion location is divided into cerebral cortex (a), subcortical areas (b), both involved (c); FMA-UE: Fugl-Meyer assessment-upper extremity.

### 3.2 Analysis of cortical activation in AMM and MTM

#### 3.2.1 Significance analysis of cerebral activation in two motor paradigms

**Figure 3:**
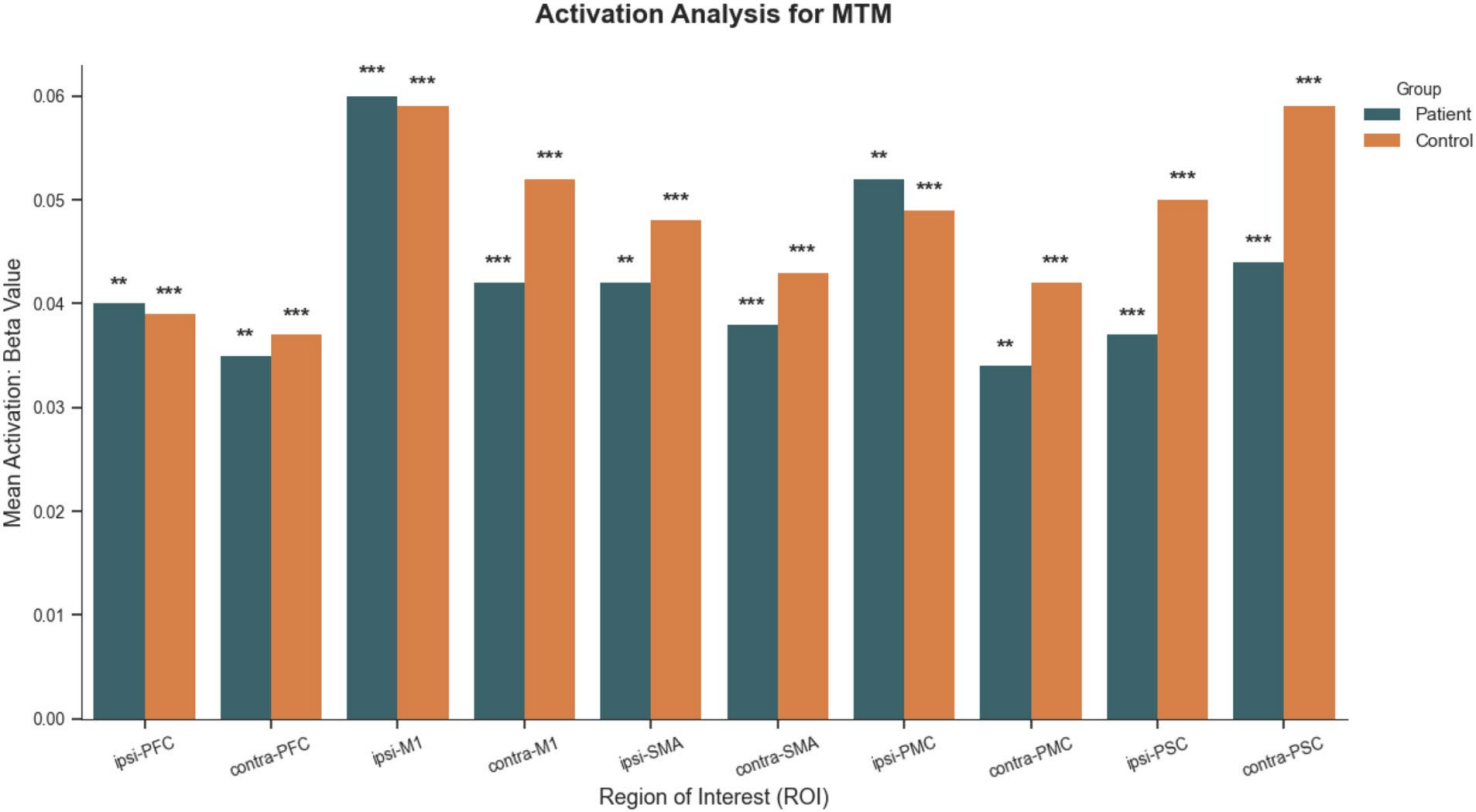
Cortical activation analysis for AMM, ipsi-: ipsilesional; contra-: contralesional; “**” represents *p* < 0.01, “***” represents *p* < 0.001.

**Figure 4:**
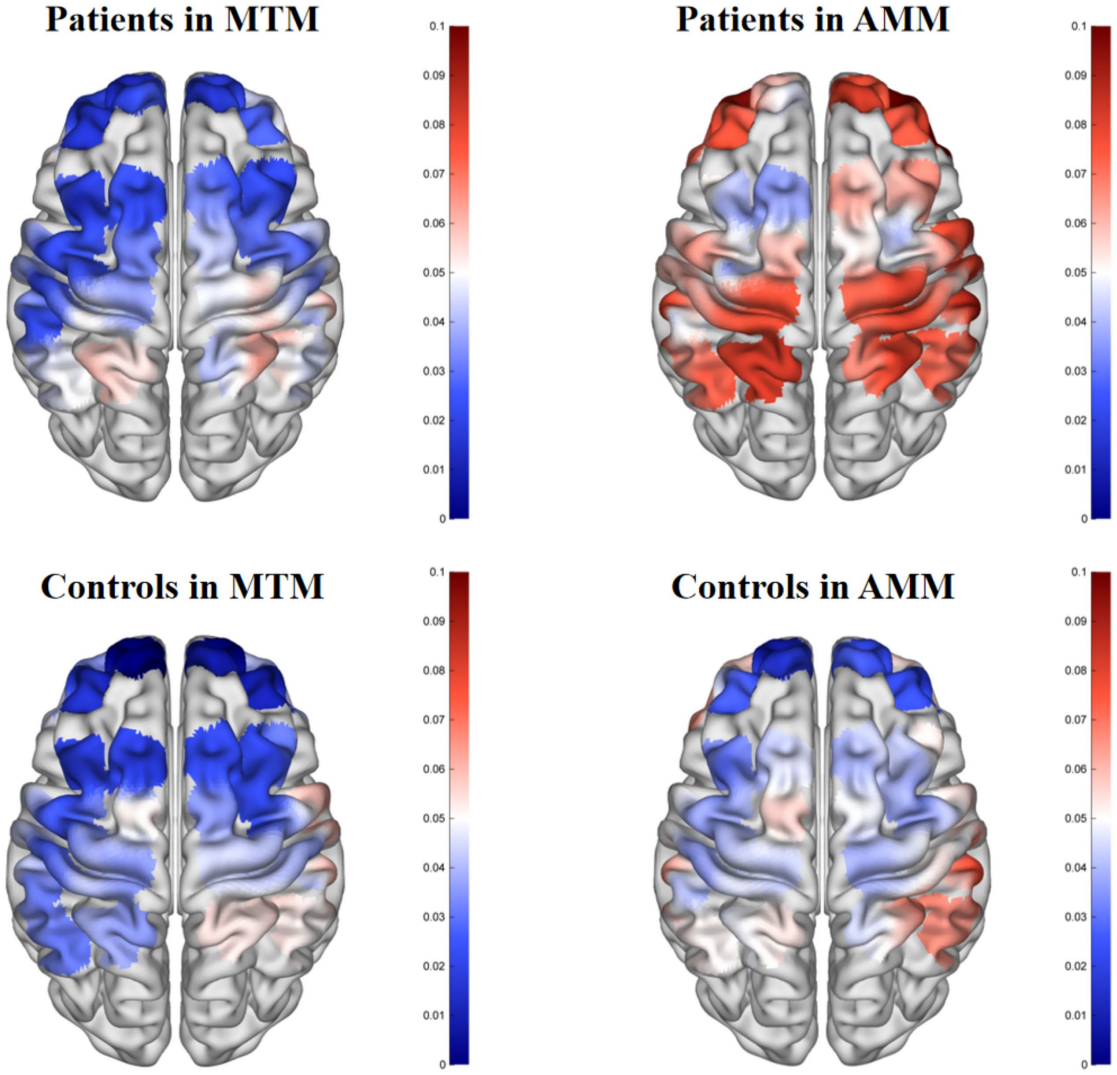
Cortical activation analysis for MTM, ipsi-: ipsilesional; contra-: contralesional; “**” represents *p* < 0.01, “***” represents *p* < 0.001.

One-sample *t*-tests or Wilcoxon signed-rank tests show that bilateral regions of interest (ROI), including the PFC, M1, SMA, PMC, and PSC, are widely and strongly activated in both stroke patients and healthy controls during active and mirror-mode robot-assisted task (RAT). As shown in Figures 3, 4, all ROIs were significantly activated in both AMM and MTM (*p* < 0.01 or *p* < 0.001, Cohen’s *d* > 0.6). These results confirm that both RAT tasks elicit hemodynamic responses well above baseline, reducing the possibility of mistaking physiological noise for neural activation and supporting the effectiveness of preprocessing steps such as motion artifact removal. Based on these findings, a two-way repeated measures analysis of variance (ANOVA) is used to examine within-group and between-group differences, along with their interaction effects.

#### 3.2.2 Cortical activation map in two motor paradigms

**Figure 5:**
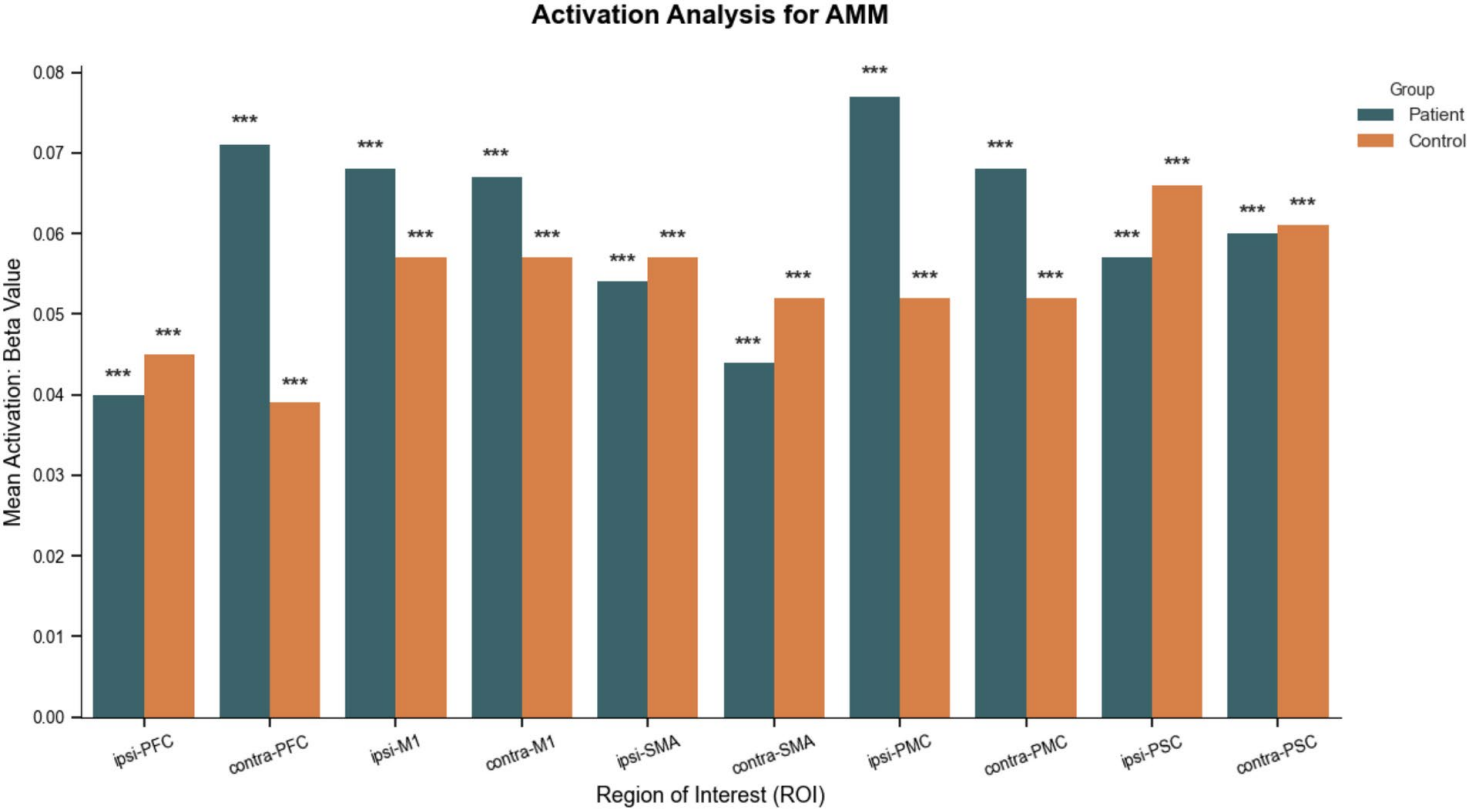
Cortical activation map of patients and healthy controls in AMM and MTM.

Topographic maps of cortical activation across groups and tasks (Figure 5) revealed distinct task-dependent patterns and significant group differences. During the AMM task, patients showed a pronounced hemodynamic response within the bilateral posterior prefrontal and sensorimotor cortices, characterized by diffuse and high activation. In contrast, healthy controls exhibited more localized activation with markedly lower intensity under the same condition. For the MTM task, both groups maintained low levels of cortical blood oxygenation, and no significant activation centers were observed.

**Figure 6:**
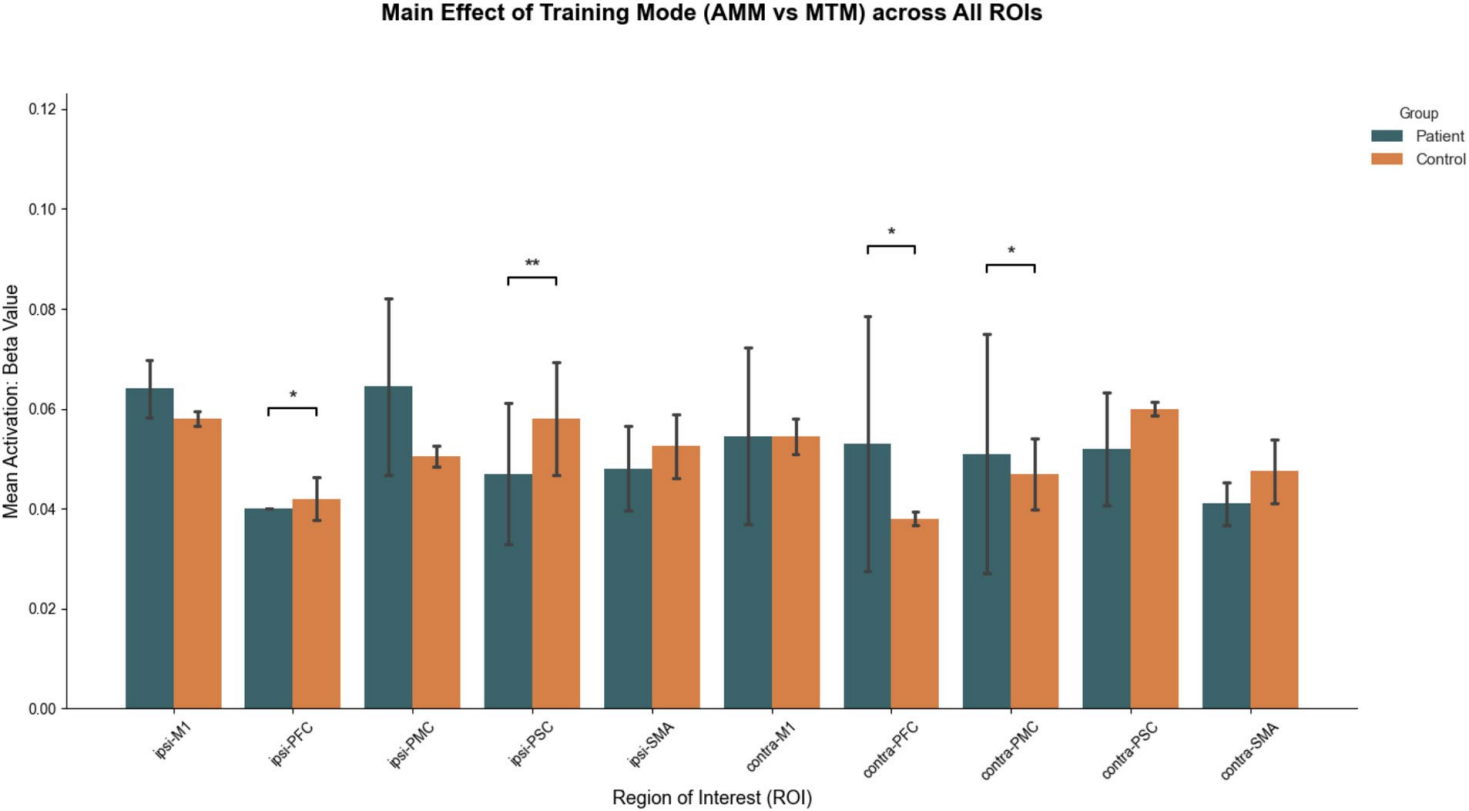
Main effect of within-group factor, “*” represents *p* < 0.05; “**” represents *p* < 0.01.

**Figure 7:**
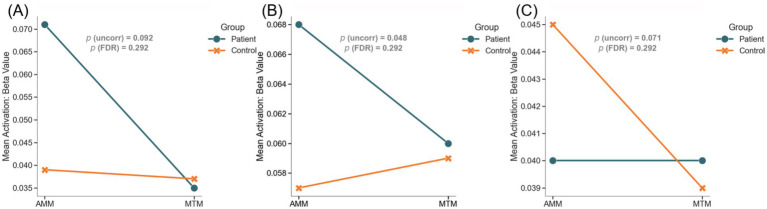
**(A)** Interaction effect of ipsi-M1; **(B)** interaction effect of ipsi-PFC; **(C)** interaction effect of contra-PFC.

**Figure 8:**
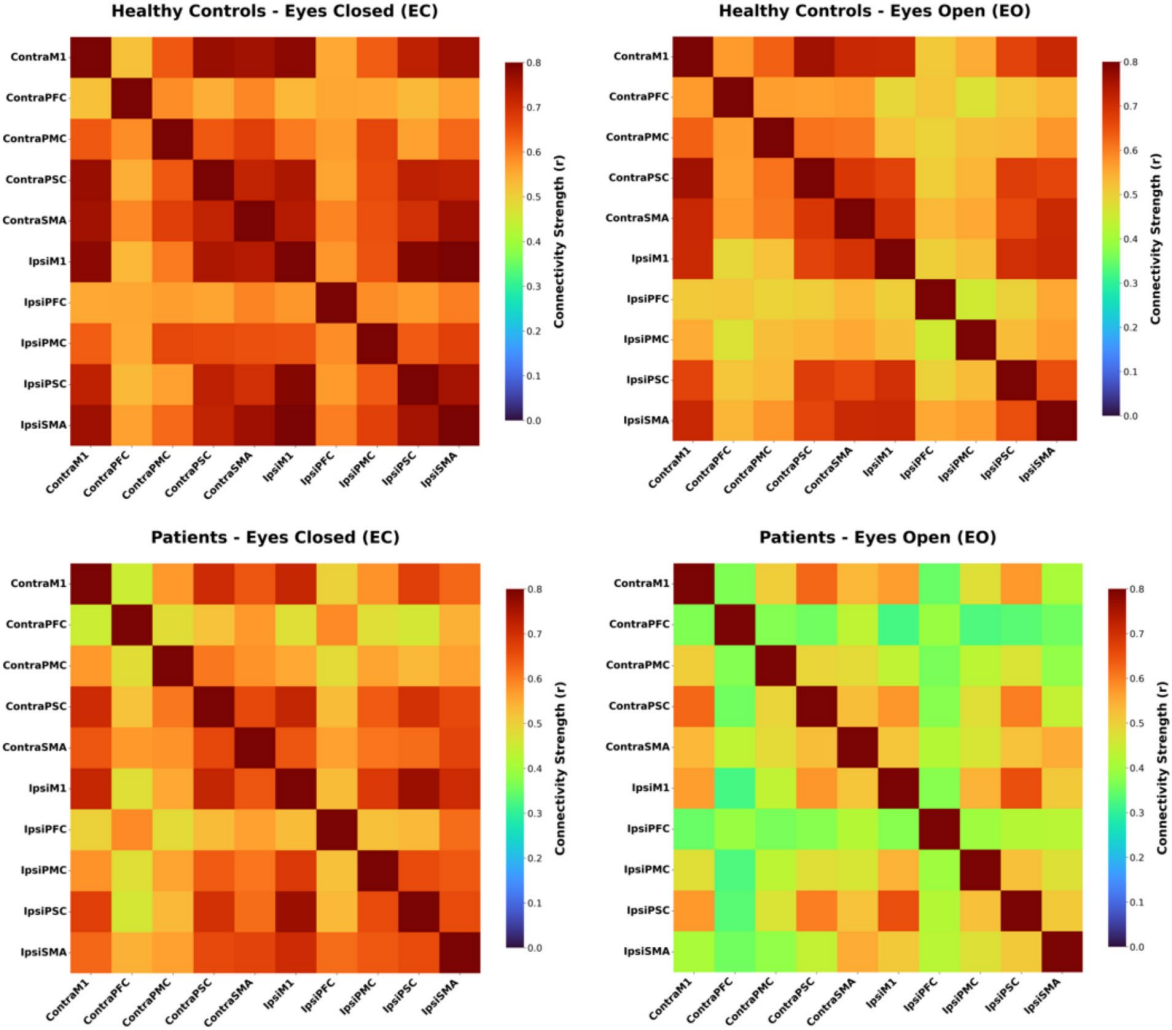
Heat map of functional connectivity of patients and healthy controls in AMM and MTM.

#### 3.2.3 Two way repeated-measure ANOVA analysis of cerebral activation

The two-way ANOVA shows significant main effects for the within-group factor in the ipsilesional PFC ( p=0.018 , $\eta^{2}$ =0.152), ipsilesional PSC ( p=0.009 , $\eta^{2}$ =0.221), contralesional PFC ( $p=0.016,\eta^{2}=0.167$ ), and contralesional PMC $(p=0.016,\eta^{2}=0.172$ ). In both groups, activation in these four regions is significantly higher in active mode than in mirror mode (Figure 6). Although the interaction effect in the ipsilesional M1 does not reach statistical significance after FDR correction ( p=0.292) , the raw significance ( p=0.047 ) and the moderate-to-large effect size ( $\eta^{2}=0.086$ ) indicate a potential clinical trend. Further analysis indicates that while patients show higher activation than healthy controls in active mode, levels are similar between groups in mirror mode (Figure 7A). Interaction effects for the ipsilesional PFC ( $p=0.292,\eta^{2}=0.072$ ) and contralesional PFC ( $p=0.292,\eta^{2}=0.063$ ) are also moderate to large but not statistically significant. Specifically, in the active mode, patients show weaker activation in the ipsilesional PFC and stronger activation in the contralesional PFC compared to controls, while mirror-mode activation remains consistent between groups (Figures 7B,C). No significant between-group main effects are found across brain regions in either task mode ( p>0.05 ).

### 3.3 Brain-behavior correlation analysis

Pearson or Spearman correlation analyses examine the relationship between regional activation and FMA-UE scores. In active mode, the contralesional M1 ( ρ=−0.370,p=0.090,p−FDR=0.906 ) and ipsilesional PSC ( ρ=−0.386,p=0.076,p−FDR=0.900 ) show a moderate negative correlation trend with FMA-UE scores, though this does not reach statistical significance (Table 3).

**Table 3:** Correlation coefficient between FMA-UE and beta value of ROIs in patients.

ROI-Mode	*r*/rho	*p*(uncorr)	*p*(FDR)
FMA-UE			
ipsi-PFC-AMM	−0.126	0.575	0.906
contra-PFC-AMM	0.182	0.418	0.906
ipsi-M1-AMM	0.067	0.766	0.906
contra-M1-AMM	−0.370	0.090	0.900
ipsi-SMA-AMM	−0.087	0.699	0.906
contra-SMA-AMM	−0.321	0.146	0.906
ipsi-PMC-AMM	−0.250	0.261	0.906
contra-PMC-AMM	0.063	0.779	0.906
ipsi-PSC-AMM	−0.386	0.076	0.900
contra-PSC-AMM	−0.171	0.446	0.906
ipsi-PFC-MTM	−0.058	0.798	0.906
contra-PFC-MTM	0.085	0.707	0.906
ipsi-M1-MTM	0.119	0.598	0.906
contra-M1-MTM	0.030	0.894	0.942
ipsi-SMA-MTM	−0.146	0.516	0.906
contra-SMA-MTM	−0.053	0.816	0.906
ipsi-PMC-MTM	0.007	0.976	0.976
contra-PMC-MTM	0.247	0.268	0.906
ipsi-PSC-MTM	−0.210	0.348	0.906
contra-PSC-MTM	−0.128	0.570	0.906

*r*: Pearson correlation coefficient; rho: Spearman correlation coefficient.

### 3.4 Lateralization analysis

One-sample *t*-tests or Wilcoxon signed-rank tests show no significant lateralization in healthy controls across modes ( p>0.05 ). In patients, the M1 region shows a trend toward ipsilesional dominance in active mode, though this is non-significant after FDR correction ( $p=0.038,p-\mathrm{FDR}=0.188,\mathrm{Co}hen^{'}s\;d=0.462$ ). In mirror mode, the PSC shows a contralesional lateralization trend that is also non-significant after correction ( p=0.045,p−FDR=0.227, $\mathrm{Co}hen^{'}s\;d=0.442$ ). No other regions show significant lateralization in either mode ( p>0.05 ; Table 4).

**Table 4:** Statistics of lateralization index (LI) across groups and modes.

Group	Mode	ROI	LI (Mean ± SD/Med)	*p*(uncorr)	*p*(FDR)	Effect size
Patient	AMM	M1	0.223 ± 0.483	0.038*	0.188	0.462
	AMM	PFC	0.028 ± 0.535	0.801	0.955	0.053
	AMM	PMC	0.054 ± 0.533	0.629	0.955	0.102
	AMM	PSC	−0.005 ± 0.429	0.955	0.955	0.012
	AMM	SMA	−0.075 (−0.868–0.268)	0.247	0.617	0.138
	MTM	M1	0.002 (−0.153–0.160)	0.731	0.731	0.008
	MTM	PFC	0.099 ± 0.519	0.368	0.460	0.192
	MTM	PMC	0.273 (−0.049–0.498)	0.119	0.259	0.404
	MTM	PSC	−0.214 ± 0.483	0.045*	0.227	0.442
	MTM	SMA	−0.190 ± 0.619	0.155	0.259	0.307
Control	AMM	M1	−0.044 ± 0.496	0.676	0.933	0.088
	AMM	PFC	0.042 ± 0.436	0.649	0.933	0.096
	AMM	PMC	0.018 ± 0.638	0.896	0.933	0.028
	AMM	PSC	0.017 (−0.042–0.165)	0.247	0.933	0.035
	AMM	SMA	−0.009 ± 0.501	0.933	0.933	0.018
	MTM	M1	0.120 (−0.264–0.195)	0.560	0.930	0.026
	MTM	PFC	−0.031 ± 0.429	0.736	0.930	0.071
	MTM	PMC	−0.012 ± 0.620	0.930	0.930	0.019
	MTM	PSC	−0.098 (−0.230–0.093)	0.300	0.930	0.032
	MTM	SMA	−0.015 ± 0.536	0.896	0.930	0.028

### 3.5 Resting-state functional connectivity analysis

One-sample *t*-tests or Wilcoxon signed-rank tests compare RSFC between groups. Mean and median values show that RSFC is lower in stroke patients than in healthy controls during both eyes-open and eyes-closed states, suggesting an overall reduction in connectivity after stroke. In the eyes-open state, patients show significantly weaker inter- and intra-hemispheric connectivity across parts of the bilateral primary motor cortex, supplementary motor area, prefrontal cortex, and primary somatosensory cortex (Table 5). No significant differences in connectivity are observed in the eyes-closed state ( p>0.05 ).

**Table 5:** Significant functional connectivity (FC) differences between groups (filtered results).

State	Connection	Patient (mean/med)	Control (mean/med)	*p*(uncorr)	*p*(FDR)	Effect size
EO	contra-M1 ~ contra-SMA	0.565 (0.401–0.721)	0.768 (0.571–0.850)	0.010**	0.049*	0.411
EO	contra-SMA ~ ipsi-SMA	0.619 (0.380–0.745)	0.781 (0.616–0.859)	0.029*	0.083	0.346
EO	contra-PFC ~ contra-PSC	0.355 ± 0.197	0.565 ± 0.136	0.000***	0.016*	−1.242
EO	contra-SMA ~ ipsi-M1	0.583 (0.339–0.689)	0.754 (0.610–0.806)	0.021*	0.072	0.368
EO	contra-PFC ~ ipsi-PMC	0.328 ± 0.208	0.472 ± 0.222	0.041*	0.109	−0.668
EO	ipsi-M1 ~ ipsi-SMA	0.536 (0.298–0.699)	0.776 (0.638–0.880)	0.012*	0.056	0.398
EO	contra-PFC ~ contra-PMC	0.371 ± 0.217	0.568 ± 0.188	0.004**	0.037*	−0.967
EO	contra-M1 ~ contra-PFC	0.366 ± 0.201	0.571 ± 0.167	0.001**	0.021*	−1.111
EO	contra-PFC ~ ipsi-PSC	0.336 ± 0.197	0.518 ± 0.161	0.003**	0.032*	−1.010
EO	contra-PSC ~ ipsi-SMA	0.424 (0.330–0.603)	0.737 (0.545–0.848)	0.005**	0.038*	0.445
EO	contra-PFC ~ ipsi-PFC	0.389 ± 0.213	0.517 ± 0.183	0.048*	0.120	−0.645
EO	contra-PFC ~ ipsi-SMA	0.371 (0.233–0.466)	0.598 (0.439–0.683)	0.007**	0.042*	0.432
EO	contra-PFC ~ contra-SMA	0.434 ± 0.204	0.569 ± 0.168	0.028*	0.083	−0.722
EO	contra-M1 ~ ipsi-PFC	0.347 ± 0.243	0.511 ± 0.198	0.025*	0.080	−0.739
EO	contra-M1 ~ ipsi-M1	0.664 (0.469–0.734)	0.826 (0.582–0.892)	0.018*	0.067	0.376
EO	contra-M1 ~ ipsi-SMA	0.408 ± 0.341	0.713 ± 0.203	0.001**	0.021*	−1.090
EO	contra-PFC ~ ipsi-M1	0.319 ± 0.197	0.488 ± 0.228	0.016*	0.067	−0.795
EO	contra-PSC ~ contra-SMA	0.525 ± 0.217	0.688 ± 0.154	0.009**	0.049*	−0.867

EO: eye open state; *p*(uncorr): *p* value before FDR correction; *p*(FDR): *p* value after FDR correction. *represents for *p* < 0.05, **represents for *p* < 0.01, ***represents for *p* < 0.001.

The functional connectivity matrices demonstrate that the patient group exhibits a widespread reduction in connectivity strength across both experimental conditions relative to the healthy control group. This observed hypoconnectivity is particularly pronounced within the circuits involving the contralesional M1 and PFC. In the eyes-open state, the healthy control group maintains a relatively stable connectivity pattern; in contrast, the patient group displays a more extensive decrease in functional connectivity, specifically within the connections associated with ipsilesional motor-related brain regions (Figure 8).

## 4 Discussion

This study provides a descriptive neurophysiological comparison of robot-assisted active movement mode (AMM) and mirror therapy mode (MTM) in stroke patients and healthy controls using fNIRS. Both AMM and MTM elicited significant task-related cortical activation across multiple motor-related regions in both groups. Compared with MTM, AMM was associated with greater activation in several regions, including the ipsilesional prefrontal cortex (PFC), ipsilesional primary somatosensory cortex (PSC), contralesional PFC, and contralesional premotor cortex (PMC). In addition, stroke patients showed weaker resting-state functional connectivity than healthy controls in the eyes-open condition, indicating altered functional organization after stroke.

### 4.1 Differences in resting-state functional connectivity and lateralization between patients and healthy controls

By including a healthy control group, this study identifies the organizational divergence of these networks during resting and task states. Analysis of resting-state functional connectivity shows that in the eyes-open condition, connectivity strength across several key brain regions is significantly lower in patients than in controls. This reduction involves inter-hemispheric and intra-hemispheric connections within the bilateral primary motor cortex, supplementary motor area, prefrontal cortex, and primary somatosensory cortex. Such widespread desynchronization suggests altered network organization after stroke and may indicate less coordinated large-scale neuronal activity. Previous research indicates that connectivity between the default mode and motor networks is impaired following a stroke, with this dysfunction correlating closely with the severity of motor impairment (21). This reduction does not reach statistical significance in the eyes-closed state, which may indicate that eyes-open conditions place greater demands on large-scale network integration. One possible interpretation is that eyes-open processing places additional demands on sensorimotor integration, under which group differences in cross-regional coordination become more apparent. This finding may be broadly compatible with prior accounts of disrupted interhemispheric balance after stroke. After a stroke, weakened inhibition from the affected hemisphere leads to hyperexcitability in the unaffected side, which interferes with recovery through abnormal contralesional inhibition (22). The reduced connectivity observed between regions such as bilateral M1 and SMA may be compatible with altered interregional coordination after stroke.

Analysis of the lateralization index further clarifies abnormalities in functional organization. In the active mode, M1 region of patients shows a trend toward ipsilesional dominance. Although not significant after multiple comparison correction, the medium effect size suggests potential relevance. Such ipsilesional compensatory activation patterns are widely reported as adaptive strategies to cope with corticospinal tract damage (23). However, the present cross-sectional data do not allow inference regarding the functional significance of this lateralization pattern. In the mirror mode, the patient PSC region exhibits a contralesional lateralization trend, while healthy controls show no significant lateralization in any region. This difference may suggest that visual-feedback tasks place different demands on somatosensory processing in patients and controls, although the underlying basis of this pattern remains uncertain. The balanced activation seen in healthy individuals confirms the flexibility of the healthy brain in processing multisensory information without over-recruiting specific hemispheres (24). Comparing healthy controls with patients thus provides a useful reference for identifying stroke-related alterations in connectivity and task-dependent lateralization.

### 4.2 Characteristics of and differences in cortical activation between patients and healthy controls during AMM task

The study also identifies distinct activation patterns during active robotic task condition. Two-way ANOVA shows significant main effects of the group factor, with the active mode inducing stronger activation than the mirror mode in both groups, suggesting stronger cortical recruitment during AMM under the present task conditions. A group-by-mode interaction effect with a medium-to-large effect size ( $\eta^{2}=0.086$ ) appears in the ipsilesional M1. Specifically, during active movement, ipsilesional M1 activation is higher in patients than in controls, whereas levels are comparable in the mirror mode. This pattern may be broadly compatible with prior observations of altered motor-system recruitment after stroke. To drive the affected limb despite altered cortical excitability, patients must recruit more neural resources, leading to higher activation levels (25). Longitudinal studies on constraint-induced movement therapy (CIMT) find that activation patterns tend to normalize as function improves (26, 27). While this study is cross-sectional, the stronger ipsilesional activation reflects the immediate state of the neural system (26). Interaction trends in the PFC show that patients have weaker ipsilesional but stronger contralesional PFC activation compared to controls. Since the PFC is central to executive function and motor planning, weakened activation may reflect lesion impact (28), whereas increased contralesional activation may indicate a different distribution of task-related control demands between groups. Meanwhile, the active mode itself imposes higher demands on attention and motor planning, which likely amplifies the divergence in PFC activation patterns between stroke patients and healthy individuals (11). Although correlation analyses are not significant, activation in the contralesional M1 and ipsilesional PSC shows a moderate negative correlation trend with FMA-UE scores, which may indicate that greater activation in these regions was associated with greater impairment severity, although these trends were not statistically significant after correction. This is consistent with the observation that functional improvement often accompanies a reduction in over-activation (29). Under the present task conditions, AMM was associated with more widespread cortical activation. Active movement recruits motor-related cortices more strongly than other modalities, which may provide stronger immediate cortical engagement (11, 28). However, the present data do not establish whether this broader activation reflects compensation, task difficulty, or other differences between the two conditions. Results show that in the active mode, activation in the PSC, PMC, and bilateral PFC is significantly stronger than in the mirror mode. This broad activation aligns with CIMT research (26). While such circumstance is common after stroke (29), our brain-behavior correlation analysis shows no significant positive correlation between the strong activation and better functional outcomes.

It is important to acknowledge, however, that the observed differences in cortical activation magnitude between AMM and MTM cannot be attributed to any single explanatory framework, given that the two paradigms differ simultaneously across multiple dimensions that each independently modulate cortical hemodynamic responses (9, 11). In brief, AMM placed greater demands on volitional effort and action agency, whereas MTM depended more heavily on mirrored visual feedback and movement of the unaffected limb (30, 31). Beyond differences in motor demand and resistance, the two paradigms also diverge fundamentally in feedback modality (proprioceptive- and effort-dominant in AMM versus visually-dominant in MTM), attentional resource allocation profile, and the degree of perceived ownership of the impaired limb’s movement—dimensions that are known to independently modulate cortical network dynamics and hemodynamic responses irrespective of overt motor output magnitude (11, 32). The greater and more widespread cortical activation observed during AMM should therefore be understood as the aggregate consequence of these combined differences in motor effort, agency, and neurocognitive context, rather than as a direct index of one paradigm’s neurological superiority or efficiency relative to the other.

### 4.3 Characteristics of and differences in cortical activation between patients and healthy controls during MTM task

In contrast to the more pronounced task-related differentiation observed during AMM, cortical activation during MTM showed a greater degree of convergence between stroke patients and healthy controls. In the present study, activation levels in core motor-related regions, including bilateral M1 and PMC, did not differ significantly between groups under the mirror mode. This relative similarity suggests that MTM, as implemented here, engaged a pattern of cortical recruitment that was less strongly shaped by the group-related differences evident during active movement (33), and the neural demands of this condition were likely distributed less toward direct voluntary generation of paretic-limb movement and more toward the integration of mirrored visual information with ongoing sensorimotor processing (34, 35).

Such an interpretation is broadly consistent with previous studies indicating that mirror-related paradigms involve a wider cortical network associated with action observation, visuospatial processing, and sensorimotor integration (7, 36, 37). Bai et al. reported that mirror visual feedback activated regions including the sensorimotor cortex, SMA, and superior parietal lobule ipsilateral to the moving hand (7), whereas other neurophysiological and neuroimaging studies have implicated the precuneus, posterior cingulate cortex, and related areas in the processing of mirror-induced movement illusion (37, 38). Against this background, the contralesional lateralization trend observed in PSC during MTM may be compatible with a greater contribution of the non-lesioned hemisphere to the processing of visually driven sensory information under mirrored conditions.

During MTM, PFC activation in patients remained comparable to that of healthy controls and lower than that observed during AMM. Relative to active movement of the paretic limb, such a pattern is consistent with the view that mirrored visual guidance may reduce demands on self-generated motor planning, executive control, and sustained attentional allocation (31). In this sense, MTM may rely relatively less on the frontal control processes required for effortful movement generation and greater emphasis on visually mediated sensorimotor coupling (33). Nevertheless, this pattern should be understood within the constraints of the present task design, rather than generalized to mirror therapy as a whole. Taken together, the principal value of current findings lies in indicating that mirrored and active paradigms may engage partially overlapping, yet differently weighted, cortical systems during upper-limb rehabilitation after stroke (11, 39).

### 4.4 Limitations and prospects

Several limitations should be acknowledged when interpreting the present findings. First, a major limitation lies in the inherent non-equivalence of the two paradigms. AMM and MTM differed not only in therapeutic mode, but also in motor effort, resistance, movement generation, feedback modality, and action agency. As a result, the observed differences in cortical activation cannot be attributed to a single mechanism. Second, the cross-sectional design captures only immediate cortical responses during task performance and does not allow inference regarding training-induced neuroplastic change over time. Third, no clinical outcomes were measured following either mode, and no significant brain-behavior relationships were observed after correction. This substantially limits the extent to which the present findings can be interpreted in functional or clinical terms. In addition, the patient cohort was heterogeneous with respect to lesion location, stroke duration, and impairment severity, which may have contributed to variability in cortical responses and may partly explain differences from other studies involving more specific stroke populations (27, 40). Finally, fNIRS is limited to superficial cortical regions and cannot directly assess subcortical structures such as the basal ganglia, thalamus, and cerebellum, all of which are relevant to post-stroke motor control and learning (41). The lack of short-separation channels is another methodological limitation, because residual extracerebral or systemic physiological contamination cannot be completely excluded despite the preprocessing procedures used.

Future studies should therefore adopt longitudinal designs to determine whether the task-dependent cortical response profiles observed here are associated with subsequent behavioral improvement or differential treatment response. More refined experimental paradigms that separately manipulate resistance, movement generation, visual feedback, and action agency would be particularly valuable for disentangling the individual contributions of these factors to cortical activation. In addition, combining fNIRS with modalities capable of probing deeper structures or broader whole-brain networks, such as fMRI, may provide a more comprehensive understanding of the neural processes underlying these rehabilitation paradigms. Such work would offer a stronger basis for assessing whether the neurophysiological differences between AMM and MTM have prognostic or translational relevance in stroke rehabilitation.

## 5 Conclusion

This study demonstrates that robot-assisted AMM and MTM are associated with distinct task-dependent cortical response profiles in stroke patients and healthy controls. AMM elicited stronger activation in selected frontal and sensorimotor regions, whereas MTM was characterized by a comparatively more convergent activation pattern across groups. Stroke patients additionally showed reduced resting-state functional connectivity relative to healthy controls, consistent with altered functional organization after stroke. These findings suggest a neurophysiological contrast between two commonly used rehabilitation modes whose cortical signatures likely reflect the combined effects of effort, resistance, movement generation, visual feedback, and action agency. Longitudinal studies incorporating behavioral outcomes will be required to establish whether these immediate cortical responses have prognostic or translational significance for stroke rehabilitation.

## Data Availability

The raw data supporting the conclusions of this article will be made available by the authors, without undue reservation.
